# Revealing Accessibility of Cryptic Protein Binding Sites within the Functional Collagen Fibril

**DOI:** 10.3390/biom7040076

**Published:** 2017-11-01

**Authors:** Cody L. Hoop, Jie Zhu, Ana Monica Nunes, David A. Case, Jean Baum

**Affiliations:** Department of Chemistry and Chemical Biology, Rutgers University, Piscataway, NJ 08854, USA; cody.hoop@rutgers.edu (C.L.H.); zhujiejz39@gmail.com (J.Z.); anamonicanunes@gmail.com (A.M.N.); david.case@rutgers.edu (D.A.C.)

**Keywords:** collagen, fibrils, ligand, interaction, solvent accessible surface area

## Abstract

Fibrillar collagens are the most abundant proteins in the extracellular matrix. Not only do they provide structural integrity to all of the connective tissues in the human body, but also their interactions with multiple cell receptors and other matrix molecules are essential to cell functions, such as growth, repair, and cell adhesion. Although specific binding sequences of several receptors have been determined along the collagen monomer, processes by which collagen binding partners recognize their binding sites in the collagen fibril, and the critical driving interactions, are poorly understood. The complex molecular assembly of bundled triple helices within the collagen fibril makes essential ligand binding sites cryptic or hidden from the molecular surface. Yet, critical biological processes that require collagen ligands to have access to interaction sites still occur. In this contribution, we will discuss the molecular packing of the collagen I fibril from the perspective of how collagen ligands access their known binding regions within the fibril, and we will present our analysis of binding site accessibility from the fibril surface. Understanding the basis of these interactions at the atomic level sets the stage for developing drug targets against debilitating collagen diseases and using collagen as drug delivery systems and new biomaterials.

## 1. Introduction

Some of the most fundamental functional fibrils of the human body are formed by collagens, which remarkably play dual structural and biological roles. Of the 28 types of collagens in the extracellular matrix (ECM), seven form higher order fibrils with a complex architecture that function in order to uphold the structural integrity of connective tissues. In addition, their interactions with molecular binding partners, such as cell receptors, enzymes, and other ECM components, are critical for the function and regulation of cellular processes [1,2,3,4,5]. The significance of collagen in cell signaling and in regulation of the ECM is high, along with its role in pathological conditions such as arthritis, cancer, and heart disease [1,2]. Collagens types I–III are the most abundant fibrillar collagens. Collagen I makes up 90% of all collagen, and it is found primarily in bone, dermis, ligaments, and cornea [1,3]. Within these tissues, protein–collagen interactions drive numerous cellular processes, yet how these very specific interactions manage to occur with either collagen monomers or fibril superstructures is not readily evident. Despite all of the incredible advances in understanding different aspects of collagen–ligand interactions through the use of collagen model peptides (CMPs) [6,7,8,9,10,11,12,13,14,15,16,17,18,19,20,21,22,23,24,25,26,27,28,29,30,31,32,33,34,35,36], the molecular determinants of the interactions between the full length collagen and its binding partners remains unclear. Here, we review known and theorized structural aspects of collagen I and discuss the binding accessibility of several critical interaction partners based on quantitative solvent accessible surface area (SASA) calculations on the smallest repeating unit (SRU) of the collagen I fibril.

## 2. The Complex Collagen Architecture 

**Figure 1:**
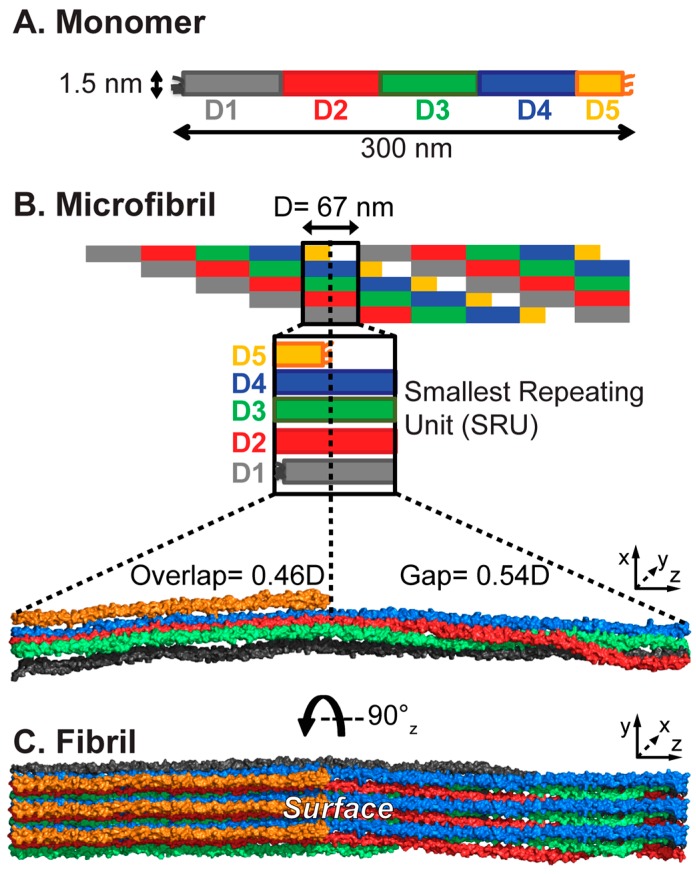
Collagen assembly from triple helical monomers to the collagen fibril architecture. (**A**) Monomer triple helix: Collagen I is a heterotrimer composed of two α1 and one α2 chains that supercoil into a long rod-like triple helix with approximate dimensions of 300 × 1.5 nm. The monomer consists of 4.46 D-periods, numbered D1–D5; D5 is 0.46D; (**B**) Microfibril: Five monomers bundle into pentameric microfibrils, in which one D-period contains the entire collagen sequence from D1 to D5. The short D5 divides the D-period into two distinct regions; the “overlap” region contains segments D1–D5, while the “gap” region is devoid of D5. This creates the characteristic D-banding pattern that can be observed by microscopy. The schematic D-band is rendered in Pymol; (**C**) Fibril surface: A 90° rotation about the long triple-helical axis reveals the fibril surface. As an example, “surface A” is shown here. For clarity, three D-periods are shown along the *y*-axis and one along the *x*-axis.

At the foundation of all collagens, there is a collagenous domain composed of repeating Gly-Xaa-Xaa^′^ sequences, where Xaa and Xaa^′^ are often proline (Pro/P) and post-translationally modified hydroxyproline (Hyp/O), respectively. Fibrillar collagens (I–III, V, XI, XXIV, and XXVII) have an uninterrupted GXX^′^ sequence. The linear collagenous sequences are over 1000 amino acids long, termed ‘α-chains’, and fold into left-handed polyproline type II (PPII) helices. Three α-chains supercoil around a common axis, staggered by one residue, to form the characteristic collagen triple helix. Within the triple helix, the Gly residues are buried on the interior, while the X and X^′^ residues are largely exposed to the solvent and are responsible for the recognition of collagen binding partners [37]. Throughout this review, the triple helix will be considered the monomeric unit. Collagen I is a heterotrimeric triple helix composed of two α1(I) and one α2(I) chains. The triple helix presents with a long, rod-like profile, with dimensions of approximately 300 nm long and 1.5 nm in diameter [37,38,39,40] (Figure 1A).

Collagen is produced as procollagen, containing propeptides in the N and C-termini. Upon secretion into the ECM, the propeptides are cleaved, exposing the telopeptides that are adjacent to the collagenous domain and initiating collagen assembly into large highly organized supermolecular fibril structures [41,42,43]. These fibrils are not amyloid in conformation, but have differently highly organized hierarchical structures. The buildup of the collagen architecture is presented in Figure 1.

Toward fibril assembly, five monomers bundle into microfibrils. Within the microfibrils, the monomers are staggered axially by 67 nm when hydrated [44]. This consistent 67 nm dimension is called the ‘D-period’ and sets the periodicity of the fibril assembly. The ≈300 nm triple helix spans the length of 4.46 D-periods and is divided into D-segments, D1–5 (distinguished by color in Figure 1), with D5 being a short 0.46D [45]. Since the monomers of the microfibril are offset by one D-period, the microfibril can be cut into units one D-period long, which contain one copy of each D-segment, D1–5, each from a different monomer (Figure 1B). This unit is the SRU, and the smallest building block of the collagen fibril. Inter-microfibrillar crosslinking between D1 and D5 stabilizes the fibril assembly [46]. Due to D5 being less than half of the length of the other D-segments, the SRU is divided into two distinct sections that are defined by their molecular densities: the ‘overlap’ region, which contains D5, and the ‘gap’ region, devoid of D5 (Figure 1B). The alternation of these regions creates the characteristic D-banding pattern of dark and light bands observed in microscopy, resulting from differential electron density (in electron microscopy) or heights (in atomic force microscopy) between the two regions. It has been established through several studies that the monomers are not stacked laterally in the microfibril, but are arranged into a quasihexagonal lattice [47,48,49,50,51,52,53]. This three-dimensional packing buries some parts of the monomers within the microfibril. From their fiber diffraction study of an intact collagen I fiber from rat tail tendon, Orgel et al. found that while in the overlap region the quasihexagonal packing is largely constant throughout its length, in the gap region, the triple helices take on a right handed twist about the fibril axis, which determines the exposure of particular domains to the accessible surface (Figure 1C) [53]. The fibril SRU contains all of these structural features, which are integrated into the larger fibril assembly, and the entire collagen I sequence for locating the binding domains within the D-period. Thus, the fibril SRU provides the simplest model to assess the accessibility of interaction domains within the collagen fibril.

## 3. Collagen Interactions and the Fibril Surface

### 3.1. Identification of Binding Sequences 

The complex assembly of monomers into microfibrils and fibrils results in one face being surface exposed and available for binding while other segments are buried within the fibril core [53,54]. Fibrillar collagens have numerous binding partners: interacting with ECM components, binding to cellular receptors, and interacting with enzymes for chemical modification and matrix metalloproteinases (MMPs) during degradation [4,55,56,57,58,59]. Despite its presentation as a long rod with a repeating GXX^′^ sequence, collagen’s binding partners recognize specific sequences and motifs across its more than 1000 residue span, and some specifically bind to particular structural conformations. Much progress has been made in determining collagen recognition sequences of cell receptors and interaction partners through the elegant use of triple helical peptides, recombinant and bacterial collagen, and sequence-dependent adhesion assays [5,60,61,62,63,64,65,66,67,68,69,70,71]. The collagen peptide toolkits developed in the Farndale laboratory are libraries of overlapping peptides that span the entire collagenous sequences of homotrimeric collagens II and III. Each peptide contains 27 residues from natural collagen sequences, including nine unique residues, flanked on each termini by five GPP repeats ((GPP)_5_) for stabilization of the triple helix [62]. Although, collagen I is a heterotrimer of two α1(I) chains and one α2(I) chain, the collagenous domain of the collagen I α1(I) chain contains 74.5% and 63.0% sequence identity to the collagenous domains of collagens II and III, respectively. Thus, these peptides provide excellent tools for identifying specific sequence motifs recognized by collagen-binding partners and comparing their relative affinities in the triple helical context.

By evaluating the binding of receptors to the collagen toolkit peptides, specific binding sequences have been determined for collagen binding integrins α1β1 and α2β1 [72,73,74,75,76], discoidin domain receptors (DDR) 1 and 2 [64,65,77], von Willebrand factor (VWF) [78,79], glycoprotein VI (GPVI) [80,81], leukocyte-associated immunoglobulin-like receptor-1 (LAIR-1) [82,83], osteoclast-associated receptor (OSCAR) [84,85], secreted protein acidic and rich in cysteine (SPARC) [86], *Yersinia* adhesion A (YadA) [87], procollagen C-proteinases enhancer (PCPE) [43], MMP1 [88], MMP13 [89], and fibromodulin [90]. Known sequences of collagen binding partners have been previously reviewed and mapped onto fibrillar collagen I and III sequences [5,56,57,91]. Due to challenges of acquiring intact monomeric collagen I, only a few studies exist on protein binding to full collagen I monomers, using microscopy techniques to detect ligand binding. The binding of heparin to procollagen type I was localized by rotary shadowing and electron microscopy (EM) [92]. Binding sites of SPARC to procollagens of types I, II, and III have been visualized by rotary shadowing EM [86] and atomic force microscopy (AFM) [93], and to the collagen I monomer lacking propeptides by AFM [93]. These experiments probe primarily protein interactions with collagen when it is in a long triple-helical rod, in which X and X^′^ residues are largely exposed and accessible for binding. The relative affinities of the recognition sequences to the collagen triple helix may not be translatable to the full fibril architecture. In the three-dimensional packing of the fibril, some of the interaction domains become “cryptic” or buried from the accessible surface [4,54,58,94,95], yet cellular functions are still carried out, and it remains difficult to understand how molecular partners recognize and interact with their specific binding motifs when packed in the three-dimensional collagen fibril.

### 3.2. Fibril Surface Identity 

The Orgel laboratory reported the identity of the solvent-exposed surface of the collagen I fibril based on fitting the X-ray fiber diffraction model of the collagen I microfibril from rat tail tendon [53] into the profile of the collagen I fibril that is observed by scanning electron microscopy (SEM) and AFM [96,97,98]. In the microscopy images, the collagen fibril follows a corrugated pattern with up-ward and downward slopes, the peak of which Orgel et al. suggests can be explained by the C-terminal telopeptide; furthermore they suggest that the C-telopeptide is exposed on the surface with the N-terminus buried within the fibril [98]. This would place segments D5 and D4 on the exterior of the fibril. Segment D5 contains a (GPO)_5_ domain adjacent to the C-telopeptide. This imino-acid rich GPO repeat is expected to form the most stable triple helix domain, and may have a protective role to less stable sequences within the fibril core [54]. However, this surface leaves buried and inaccessible high affinity recognition sequences for collagen-binding integrins and the single interaction site for platelet receptor VWF, which is shared with DDRs and SPARC. Herr and Farndale have proposed an alternative orientation with the opposite face exposed that would make these sites more accessible [58]. This raises an interesting question: if critical interaction domains are buried within the collagen fibril interior, how do biological processes that depend on these interactions occur? In order gain a perspective on the residue-specific exposure of identified binding motifs within the collagen fibril, we used computational tools to build a collagen fibril model and calculate SASA from the fibril surface of each residue in our model.

## 4. Computational Studies of the Collagen Fibril 

Streeter and de Leeuw first built an all-atom model of the collagen fibril based on the atomic structure and periodic nature of the fibril, as reported by Orgel (Protein Data Bank (PDB) entry: 3HR2) [53], and performed molecular dynamics (MD) simulations on the collagen model with periodic boundaries to replicate the supermolecular arrangement [99]. The simulation was validated by comparison to experimental observations and theories of the collagen fibril structure. Streeter and de Leeuw also studied the inter-protein interactions within a collagen fibril [100]. Simulations of collagen fibrils and monomers were compared to study the atomic level interactions upon fibrillogenesis. They also compared the process of collagen fibrillogenesis with the process of protein folding and concluded that they both act to shield hydrophobic side-chains from solvent, and they differ in the percentages of major intra-protein hydrogen bond types (e.g., backbone–backbone, backbone–side-chain, and side-chain–side-chain) [100].

Varma and Schieber also performed MD simulations on collagen models with periodic boundaries, and evaluated the effect of several factors on D-band shrinkage [101]. They found that the large shrinkage that was observed in earlier simulations was not eliminated by optimizations of temperature/pressure coupling algorithms, salt concentration or hydration level, or the cross-linking of monomers, and concluded that the shrinkage was a force-field artifact. A new force field Amber99sb-ildn was found to produce a small D-band shrinkage of <3% [101].

Here, we aimed to create a measure of accessibility for known specific receptor and protein binding sequences within the collagen I fibril. Previously, Orgel et al. created a qualitative ranking of accessibility of collagen binding sites based on their distance from and relative occlusion by the fibril surface [54] and calculated SASA within the fibril to evaluate accessibility of the MMP cleavage site [98]. We have now used a modified SASA method to characterize the ability of a binding partner to access its specific binding site within the collagen fibril SRU that is nearest to the fibril surface.

### 4.1. Building the Atomic Model of the Collagen Fibril 

**Figure 2:**
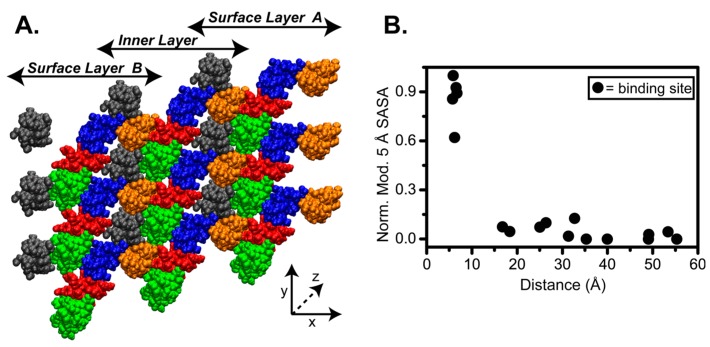
(**A**) A representative cross-section showing the 3 × 3 SRU matrix used in the solvent accessible surface area (SASA) calculations. The model contains three SRUs in each the *x*- and *y*-dimensions, and the *z*-dimension extends to one D-period in length. Triple helical monomers are color-coded by D-segments as in Figure 1. Double-headed arrows indicate the three distinct layers in the x-dimension. (**B**) Correlation of SASA with binding site distance. The modified 5 Å SASA of 17 known binding sites with respect to the orientation proposed by Orgel et al. (“surface A”) is plotted vs. distance of the site from the surface.

We have constructed an atomic model of a collagen fibril unit cell based on the low-resolution fiber diffraction structure (PDB entry 3HR2 [53], see Figure 1 for fibril architecture). The fiber diffraction provided structural information about the Cα atoms, giving the overall shape and orientation of the collagen monomers. From this structure, we built a complete all-atom monomer model by adding side-chain and backbone atoms, plus water molecules to fill the repeating unit cell, following work by the Leeuw group [99,100]. This was equilibrated by MD simulations with loose tethering of the Cα atoms to their positions in the 3HR2 structure [53]. Five collagen triple helix monomers from the equilibrated model were duplicated and packed by D-period to create a microfibril and then cut along the z-axis to the length of one D-period to generate one SRU. A finite, non-periodic fibril SRU model was then built with one complete D-period along the *z*-axis, and three SRUs along the *x*- (depth) and *y*- (height) axes (Figure 2A). Unlike periodic models, this fibril model has a surface on either side, surface layer A (with D5 and D4 on the surface) and B (with D1 on the surface), where proteins or other effectors may bind, and an inner layer that is completely shielded from a fibril surface.

### 4.2. SASA of Collagen I Fibril Reveals Exposure of Partner Binding Sites from the Fibril Surface 

In order to assess the site-specific accessibility at the collagen fibril surface, we performed SASA calculations with different probe sizes on all residues of the 3 × 3 fibril SRU model (Figure 2A) using the Molecular Surface (MS) program [102,103]. In these calculations, the SASA gives the surface area of an individual residue that is accessible to a probe of a certain radius. We initially calculated SASA with a 1.4 Å probe, the radius of a water molecule, in order to measure the water accessibility within the fibril. Remarkably, there is uniformly high SASA, even into the D-segments of the inner layer, to water molecules throughout the 3 × 3 fibril SRU model. This indicates that despite appearing densely packed, there is significant space for water within the triple helices, even within the core of the collagen fibril.

In an attempt to model access of ligands to the binding sites in the fibril, we determined the SASA of the different D-segments, using a larger spherical probe with a 5 Å radius, termed the 5 Å SASA. As we are interested in determining binding site exposure relative to the surface A or surface B (see Figure 2A), we used a simple approach in which we subtract the 5 Å SASA of SRUs of the inner layer from the 5 Å SASA of the surface layer. This subtraction gives accessibility only from the fibril surface, when compared to the fibril core. This allows us to probe the accessibility of the residues that are exposed to the fibril surface. We applied the calculation to each residue of the middle SRUs along the *y*-axis of the 3 × 3 fibril SRU model (Figure 2A) since they are surrounded by all of the neighbors that would be present in the full fibril in order to obtain a baseline for the 5 Å SASA. As the triple helix is composed of three chains, we used the average 5 Å SASA of the three chains at each position for the calculation.

We have plotted the modified 5 Å SASA of 17 known binding sites in collagen I with respect to their distance from the surface A, which exposes D4 and D5 (Figure 2B). We find that of these 17 sites, only five show a high modified 5 Å SASA; the other sites appear to have minimal modified 5 Å SASA suggesting that the majority of the collagen binding sites are not accessible from surface A. This underscores that although access to these binding sites by binding partners is required for a multitude of biological processes, their exposure within the context of the collagen fibril is minimal.

**Figure 3:**
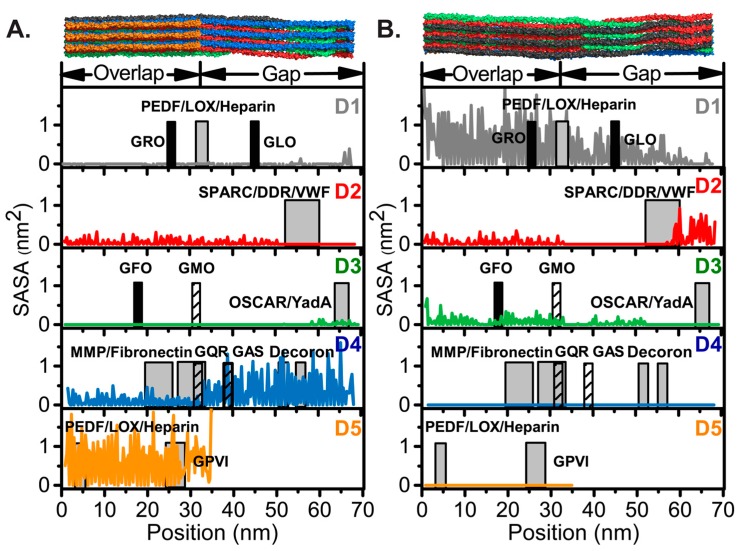
Residue-specific modified 5 Å SASA calculations with respect to surfaces (**A**) A [98] and (**B**) B [58]. The D-segments, D1–D5, are indicated and colored as in Figure 1 and Figure 2. The locations of collagen ligands are shown by gray boxes with heparin [104], PEDF [105] and LOX [57] in D1 and D5; SPARC [86], DDRs [64,65] and VWF [78] in D2; OSCAR [84,85] and YadA [87] in D3; MMPs [68], fibronectin [70], and the decorin core protein, decoron [106], in D4; and GPVI [80] in D5. Integrin high and low affinity motifs are represented by black and hashed boxes, respectively, and labeled by the first three residues of the indicated six-residue binding motif. The width of the boxes corresponds to the length of the identified recognition sequence. Abbreviations: PEDF, pigment epithelium-derived factor; LOX, lysyl oxidase; SPARC, secreted protein acidic and rich in cysteine; DDRs, discoidin domain receptors; VWF, von Willebrand factor; OSCAR, osteoclast-associated immunoglobulin-like receptor; Yad A, *Yersinia* adhesin A; MMPs, matrix metalloproteinases; GPVI, glycoprotein VI.

We analyzed the modified 5 Å SASA in residue-specific detail throughout the collagen fibril. For visualization purposes, we present SASA form the perspectives of both surfaces A and B. The residue-specific modified 5 Å SASA for each D-segment relative to a particular surface is plotted in Figure 3A,B. Our SASA analysis reveals non-uniform SASA between the D-segments, as expected due to the quasihexagonal arrangement of the microfibril. In particular, high SASA is observed in the D-segment at the assumed surface (D4 and 5 in surface A and D1 in surface B), and the accessible surface area is significantly lower in D-segments deeper into the fibril. Moreover, the SASA is not uniform within each D-segment along the length of the D-period due to the inherent twisting of monomers within the fibril (see schematic at the top of Figure 3A,B). For example, from surface A, D2 has moderate accessibility in the overlap region and in the N-terminal half of the gap region, but no accessibility in the C-terminal end of the gap region (Figure 3A). The twisting out of the D3 at this end of the gap makes it lowly accessible, and it is non-accessible in the remainder of the D-period. Conversely, surface B gives moderate accessibility to the overlap region and the N-terminal half of the gap region of D3, and moderate accessibility of D2 in the overlap region (Figure 3B). In this orientation, the twisting out of D2 at the C-terminal end of the gap region gives it higher accessibility than even D1, which is at the surface and has the highest exposure for most of the D-period. The variability of SASA both between D-segments and along the D-period makes the exposure of binding sites complex.

The accessibility from the fibril surface is highly determinant of interactions with molecular partners. We therefore analyzed the accessibility of thirteen collagen-binding ligands from both surfaces A and B (Figure 3). The SASA highlights the highly accessible binding sites near the surface in either orientation. Quantification of the residue-specific SASA of the 3 × 3 collagen fibril SRU model in both possible orientations provides a measure of accessibility of interaction domains when either face is exposed.

Presentation of the C-terminus to the surface provides direct access to essential fibril interaction sites, including lysyl oxidase (LOX) [57], GPVI [80,107], and decoron, the core protein of decorin [106]. LOX interaction sites are on both D1 and D5 for formation of intermolecular cross-links that regulate the collagen fibril assembly. LOX deaminates lysines and hydroxylysines in preparation of cross-link formation between triple helices to stabilize collagen fibrils [108]. GPVI is a glycoprotein on platelet membranes that interacts with collagen to activate the platelets and induce platelet aggregation [107]. GPVI recognizes and binds to short (GPO)_n_ tandem repeats on collagen. The only GPO repeat motif longer than (GPO)_2_ in the collagen I sequence is in D5 at the very C-terminus of the collagenous domain and contains (GPO)_5_ in the α1(I) chain and (GPO)_4_ in the α2(I) chain. Decorin is a proteoglycan that interacts with collagen I fibrils to stabilize the suprafibrillar assembly [106,109]. The core protein of decorin, decoron, recognizes two binding motifs in the gap region of D4, KXGDRGE, and AKGDRGE [106,109]. An X-ray crystal structure of decoron has been solved and docked onto the collagen I fibril when D4 is exposed [106]. Access of these sites in the mature fibril of collagen would allow these proteins to carry out their cellular functions.

Furthermore, in this orientation there is a potential means of collagen fibril cleavage by MMPs. MMPs are the only enzymes that are capable of cleaving collagen molecules. Degradation of collagen fibrils is essential for fibril turnover, tissue repair, and development. MMP1, MMP8, MMP13, and membrane type-MMP1 (MT-MMP1) cleave collagen α-chains at a single, specific Gly-Ile/Leu cleavage site, dissecting the triple helix into three-quarter and one-quarter length fragments. MMPs have been proposed to cleave one α-chain at a time, starting specifically with the α2 chain in collagen I [110,111]. The MMP cleavage site is located in the overlap region on segment D4, and therefore is accessible upon displacement of the C-telopeptide. In an *in silico* study, Perumal et al. proposed how collagenolysis can occur in the collagen fibril. From the C-terminal fibril surface, the α2 chain is oriented toward the outside of the fibril and has a greater dissociation from the center of the triple helix, making it more vulnerable to MMP cleavage [98]. Moreover, the protection of the site of collagenolysis by the C-telopeptide increases MMP specificity, as it will only be able to cleave when the C-telopeptide is displaced [98,112]. Degradation of the collagen fibril is known to occur, and thus access of MMPs to their cleavage site is essential.

On the other hand, surface B favors access to high affinity integrin binding motifs and platelet receptor recognition sites. In this orientation, known platelet interaction sites, including those for integrin α2β1 and VWF, are more accessible to their binding partners. Both integrin α2β1 and VWF have been shown to be critical receptors for the platelet adhesion to collagen [55,113]. Many specific binding sequences for platelet and other cell receptors have been determined from adhesion assays to fully exposed triple helical collagen II and III peptides. Moreover, Jokinen et al. showed that through solid-phase cell adhesion assays and immunoelectron microscopy, that integrin α2β1 does in fact adhere to mature collagen I fibrils from bovine skin and facilitates cellular responses, such as cell spreading and formation of cellular projections [114]. This suggests that the integrin α2I domain must have access to at least one of its recognition domains on the collagen I fibril. In which sequence the integrin α2I actually binds in collagen I fibrils has yet to be determined.

In Figure 3, black and hashed boxes indicate integrin high and moderate affinity binding motifs, respectively. High affinity integrin α2β1 binding sequences, GROGER and GLOGER [58,62,74,76,115], are on D1 and would be directly on the surface of the collagen I fibril available for α2I domain binding. High affinity integrin binding site GFOGER and moderate affinity binding site GMOGER, on the N-terminal half of D3 also become more accessible with surface B exposed, allowing accessibility to all high affinity integrin α2I binding sites. Furthermore, only in this orientation is it possible to gain accessibility to the single VWF binding motif, GVMGFO, on the C-terminal end of D2, which is overlapped with the binding sequences for SPARC and DDRs [64,65,78,86].

Although surface B provides more access to high affinity integrin binding motifs and the GVMGFO motif shared by VWF, DDRs, and SPARC binding partners, this surface does not allow access to the MMP cleavage and binding sites or GPVI and decoron interaction motifs. Yet, integrin binding from surface A is not ruled out, as moderate affinity binding motifs, GQRGER and GASGER, are accessible from this surface, especially if the C-telopeptide is dynamic. The dynamics of the C-telopeptide and proteolysis by MMPs might provide access to functional domains otherwise buried within the fibril, and would therefore support the surface exposure of the C-terminus, as proposed by Perumal et al. [98], rather than the N-terminus. Considering the accessibility of critical fibril binding sites, as assessed by our SASA analysis, and the structural features observed by microscopy, we propose that surface A, that proposed by the Orgel laboratory, provides greater access to critical binding motifs.

## 5. Perspective

### 5.1. The Collagen Architecture as a “Smart Fibril”: Regulating Accessibility of Partner Binding Sites

The surface proposed by Orgel et al., placing the C-telopeptide on the fibril surface seems to be more accommodating of essential molecular interactions within the collagen fibril. The placement of the MMP cleavage site beneath the C-terminal D5 allows for the dynamics and motions of D5 to act as a gatekeeper, regulating the accessibility of the MMP binding and cleavage site [94]. MMP cleavage of the collagen fibril is critical for fibril turnover, tissue repair, and development. From the opposite perspective, this site would be buried deep within the fibril and be inaccessible for collagenolysis. Once degraded by MMPs, new interaction sites would become available in the cleaved collagen.

With the C-telopeptide exposed, high affinity integrin binding sites are not accessible. However, the relative binding affinities for integrins were determined in fully exposed, triple helical peptides, and may not be translatable to the context of the complex assembly of the collagen fibril. Constant strong binding of integrin to the collagen fibril may not be optimal. Integrins aid in cell adhesion and movement along collagen, and if bound too tightly, cell movement may not be possible. It has been hypothesized that this tight regulation is biologically important as it limits platelet aggregation under “healthy” conditions but promotes firm platelet adhesion upon cellular activation caused by disruption of blood vessels [55,58,116,117]. Using a ligand accessibility ranking system, Orgel et al. predicted integrin binding sites to be “cryptic”, buried within the fibril assembly, and only partially accessible in the mature fibril [54]. They proposed that cell-collagen interactions are regulated by binding motifs being exposed only upon structural reorganization or degradation of the fibril [54]. Although the high affinity integrin-binding motif, GFOGER, on D3 is buried deep within the fibril, it may be accessible upon limited proteolysis or during fibril assembly [54,94,98].

Of course, fibrils of different collagen types may have very different microfibrillar arrangements and binding interfaces. Relative affinities of integrin binding motifs and other interaction sequences of collagen binding partners have been largely determined via triple helical peptide binding studies on collagen II and III sequences. While these collagens share a high percentage of sequence identity, the similarities in arrangement into fibrillar structures have yet to be determined. A recent study by Woltersdorf et al. reported on the adhesion of collagen-binding integrins in chondrocytes to a 8:1:1 mixture of collagens II, IX, and XI mimicking cartilage collagen [118]. They found that the chondrocytes adhered to triple helical collagen, but were bound only weakly to collagen in the fibrillar form. By immunoelectron microscopy, they observed recombinant integrin I-domains binding not directly to the collagen fibrils, but to residual non-fibrillar material peripheral to the fibrils [118]. While a previous study had observed the binding of integrins to collagen I fibrils, this recent study suggests that in cartilage collagen fibrils composed of collagens II, IX, and XI, integrins do not have access to their recognition sites.

### 5.2. Regulation of Protein Binding through Internal Dynamics of the Collagen Fibril

The SASA calculation assumes only a snapshot of a configuration of the fibril in time. The fibril and the monomers within are not static entities, but are constantly undergoing internal motions. These fluctuations could provide a key to understanding how binding partners are able to access their seemingly buried binding sites. For example, from the surface proposed by Orgel, the MMP cleavage site is buried beneath the C-telopeptide. Although from the SASA, this domain is only moderately accessible, backbone motions of the C-telopeptide may create an opening for an MMP to proteolyze at the cleavage site. Likewise, from the orientation, an active binding site on D2 for SPARC, DDRs, and VWF are hidden by the D1. However, the backbone dynamics of D1 in this region may reveal a path of access for these binding receptors. Proteins are dynamic in nature, and therefore, their motions may have large impacts on their capabilities to function. Future studies of internal dynamics of the collagen fibril and ligand binding will deliver new insight about how interaction partners access their specific recognition motifs and the functionality of fundamental collagen fibrils.
